# On the validity of popular assumptions in computational drug design

**DOI:** 10.1186/1758-2946-3-S1-O18

**Published:** 2011-04-19

**Authors:** Gerhard Klebe

**Affiliations:** 1 Inst. of Pharmaceutical Chemistry, University of Marburg, Marbacher Weg 6, 35032 Marburg, Germany

## 

Computational methods such as 3D-QSAR, docking, virtual screening or structure-based de novo design have been established over the last years as powerful and frequently consulted tools in lead discovery and development. As a matter of fact these methods are approximate, sometimes due to reasons to keep them computationally tractable, sometimes simply because we have not yet understood the involved complexity better, sometimes because we start from false or inappropriate simplifications and sometimes because we believe that higher accuracy of our considerations is not really required. To move the quality of our tools forward it is therefore appropriate to reflect and validate popular assumptions usually applied in computational approaches. Validation can only be performed with respect to an agreement with experimental evidence or in some cases highly sophisticated computational studies that will not be possible on a routine base.

One popular concept acts on the assumption that a bound ligand must exhibit perfect shape complementarity with a given binding pocket to achieve high affinity binding. However, high affinity corresponds to a strongly negative free energy of binding which can result from either enthalpic or entropic effects. Thus, also ligands with high residual mobility and pronounced flexibility can experience strong binding even though their actual shape complementarity with the binding pocket is far from perfect.

To keep things simple, additivity of functional group contributions is a very popular concept in structure-based drug design. Many scoring functions built on this assumption, QSAR exploits this idea and medicinal chemists love this conception to plan the optimization of their drug molecules. However, cooperativity of molecular interactions can easily lead to the breakdown of simple additivity rules.

It is well known that many of our force-field based approaches consider better the enthalpic contribution to binding than the entropic portion. Therefore, one can frequently find the hypothesis that within a congeneric series of closely related ligand analogs the entropic contribution to binding is assumed to be equivalent. This very compliant argument turns out to be a rather arbitrary assumption usually not valid even in close congeneric series due to enthalpy-entropy compensation phenomena.

Very pragmatically the protonation states of functional groups of either residues or candidate ligands are set to standard values. However, such doing neglects the fact that strong shifts of pKa values can occur which perturb the protonation states and even shift ″normal″ into charge-assisted H-bonds.

Water is an essential partner in ligand binding. Replacement of water from the uncomplexed binding pocket is assumed to be entropically favorable. However, depending on the degree of disorder of such water molecules prior to their replacement the thermodynamic signature of the water release (hydrophobic effect) can range from entropic to enthalpic. Furthermore, over again congeneric ligands can differ in their thermodynamic signature whether they release or pick-up a certain number of water molecules upon binding.

